# On the primer binding site mutation that appears and disappears during HIV and SIV replication

**DOI:** 10.1186/s12977-015-0201-5

**Published:** 2015-08-25

**Authors:** Ben Berkhout, Atze T. Das

**Affiliations:** Department of Medical Microbiology, Laboratory of Experimental Virology, Center for Infection and Immunity Amsterdam (CINIMA), Academic Medical Center, University of Amsterdam, Meibergdreef 15, 1105 AZ Amsterdam, The Netherlands

## Abstract

A recent study by Fennessey et al. (Retrovirology 12:49, 2015) described the optimization of a popular SIV clone by removal of four suboptimal point mutations. One of these mutations is present in a non-coding part of the viral genome and is probed in that study in more detail because of some fascinating properties. This primer binding site (PBS) mutation reverts rapidly to the wild-type sequence, which the authors interpret as indicating that this mutation exerts a profound fitness impact. The authors proposed the involvement of a cellular DNA repair mechanism in the reversion. Furthermore, it was suggested that premature termination of reverse transcription can explain why some of the viral progeny still contained the mutant sequence. However, we argue that all these special properties are a direct consequence of the unique nature of the viral PBS motif. The PBS binds the tRNA primer for reverse transcription and the viral progeny inherits either the sequence of the cellular tRNA or the PBS sequence of the viral RNA genome. The presence of a variant tRNA species explains the rapid appearance and disappearance of a variant PBS sequence.

## Optimization of the SIV clone by PBS adaptation

SIVmac239 is a frequently used prototype virus in HIV-1 transmission, pathogenesis and vaccination studies in the SIV-macaque model. Early studies identified four suboptimal and therefore genetically unstable nucleotides in the molecular clone encoding this virus [1]. These four suboptimal nucleotides are present in three genes encoding the reverse transcriptase (RT), integrase (IN) and envelope (Env) proteins and in the primer binding site (PBS), a regulatory sequence in the non-protein-coding leader of the HIV–SIV genome that is involved in the process of reverse transcription. Fennessey et al. recently reported in *Retrovirology* the construction of the SIVmac239Opt clone with all four positions repaired, which could form an improved virus reagent for specific studies, e.g. when using a low multiplicity of infection to challenge macaques [2]. Then the authors focus on the rather intriguing PBS mutation (T instead of C at position 8 in the 18-nucleotide PBS sequence) because the mutant PBS reverts with unparalleled speed. In fact, this PBS mutation was suggested early on to affect the virus replication potential [3], and Fennessey et al. continued along this theme of a fitness impact based on the rapid reversion kinetics [2]. First, they cultured SIVmac239 on SupT1-R5 cells for 2 months and documented a complete PBS reversion at day 21. In comparison, Env reversion occurred at approximately week 10 and no RT and IN reversions were observed within 2 months. Based on these results, the authors conclude that the PBS mutation has the greatest impact on viral replication and that reversion to the wild-type PBS sequence results in a large fitness gain. Single genome amplification was used to document the rapid reversion in more detail: 10 % reversion was scored after just 12 h and 50 % within 8 days. It was concluded that reversion of the suboptimal PBS nucleotide occurs at a rate of 5 % per day, consistent with a profound fitness impact. The authors went on to show that such quick repair occurs even in a single cycle infection experiment, that is in the absence of any virus replication, and they suggest the involvement of host DNA mismatch repair mechanisms. Furthermore, they suggest premature termination of reverse transcription as a possible mechanism for the sustained presence of proviruses with a mutant PBS.

## The PBS and tRNA^Lys^ variants used for reverse transcription

The same PBS mutation has repeatedly popped up in literature in a diversity of HIV–SIV studies, e.g. virus mutation-reversion studies [4, 5]. Its special character in terms of rapid repair has spurred several exciting, but not necessarily correct mechanistic explanations. For instance, Yu et al. attributed this mutation to the action of the cellular cytidine deaminase APOBEC3G [6]. This suggestion seemed strange as the SupT1 cells used lack this editing enzyme and consequently are permissive for a ∆vif HIV-1 mutant [7]. Based on mechanistic insight into the process of initiation of HIV-1 reverse transcription, we earlier proposed an alternative scenario that provides a rather simple explanation for this series of exotic scenarios [8, 9], including the suggested large fitness effect of the PBS mutation, the proposed DNA repair and the aberrant reverse transcription pathway proposed by Fennessey et al. [2].

We and others probed the exclusive usage of tRNA^Lys3^ as primer for reverse transcription by mutating the complementary 18-nucleotide PBS in the HIV-1 genome [10–13]. The 18 nucleotides at the 3′ end of the tRNA primer anneals to the PBS. In a complex reverse transcription process, this tRNA sequence is copied into the plus-strand of the proviral DNA, while the PBS sequence is copied into the minus-strand. Strand separation and duplication during subsequent cell division will result in proviral DNAs with the PBS corresponding to either the original viral sequence or to the tRNA primer sequence. As a consequence, the PBS sequence may tell us which tRNA primer was used, as exemplified by the reversion patterns of the set of PBS mutants that called upon another tRNA species [10, 14, 15]. The important role of the PBS in reverse transcription explains the high, but not absolute conservation of this sequence among virus isolates. Most HIV–SIV isolates have a PBS that is perfectly complementary to the 3′ end of the tRNA^Lys3^ primer (PBS^Lys3^) and this segment is among the best conserved parts of the retroviral genome. However, we reported a single nucleotide polymorphism in around 5 % of natural HIV–SIV isolates and argued for the infrequent use of a variant tRNA primer [8]. Candidate tRNA^Lys5^ molecules that encode this PBS-mutation (PBS^Lys5^) were identified when the sequence of the human genome became available [9]. We used a tRNA primer identification assay to demonstrate that tRNA^Lys3^ initiated reverse transcription at PBS^Lys3^ in 81 of 90 virus particles analysed, but the variant tRNA^Lys5^ was used in the other 9 particles (10 %) [9]. Once reverse transcription is completed, the double-stranded DNA will integrate into the host cell genome. In case of imperfect complementarity (tRNA^Lys5^ priming on PBS^Lys3^ and tRNA^Lys3^ priming on PBS^Lys5^), a heteroduplex PBS is generated with the plus-strand sequence copied from the tRNA primer and the minus-strand copied from the viral RNA. It cannot be excluded that this duplex is a target for mismatch repair as described in [2]. However, this seems less likely as Berwin and Barklis demonstrated that the plus and minus strands of an integrated retroviral heteroduplex PBS are copied and separated during DNA replication before mismatch correction can occur [16]. In this way, cell division will result in the production of proviruses with either PBS^Lys3^ or PBS^Lys5^ at a 50–50 ratio. Thus, 10 % tRNA^Lys5^ priming would result in 5 % PBS^Lys5^-containing proviral genomes, which is strikingly similar to the frequency in natural HIV–SIV isolates and the 3 % frequency that we reported in HIV-1 evolution studies [8].

## The tRNA primer dictates the PBS sequence of the viral progeny

In their single genome analysis of proviruses upon infection with a PBS^Lys5^ HIV-1, Fennessey et al. demonstrate that approximately 50 % of the proviruses contain a (homoduplex) PBS^Lys5^ sequence. This result can be explained by the use of tRNA^Lys5^ for initiation of reverse transcription. The other 50 % of the proviruses contained either a PBS^Lys3^/PBS^Lys5^ heteroduplex (40 %) or the reverted PBS^Lys3^ sequence (10 %), both resulting from tRNA^Lys3^ annealing (before and after DNA duplication, respectively). Thus, tRNA^Lys3^ and tRNA^Lys5^ anneal with similar efficiency to PBS^Lys5^ (~50 %), whereas tRNA^Lys3^ binds with higher frequency than tRNA^Lys5^ to PBS^Lys3^ (90 and 10 %, respectively, as described above). As a consequence, the PBS^Lys5^ variant percentage will gradually decrease further upon subsequent rounds of virus replication and cell division, while the PBS^Lys3^ fraction will increase. No matter the PBS composition of the starting clone, HIV–SIV will soon replicate as a mixture of roughly 95 % PBS^Lys3^ and 5 % PBS^Lys5^ variants, which are no stable entities but rather interchange continuously, but maintaining this fixed 95–5 ratio. In other words, this PBS-variation is driven by the tRNA primer usage and occurs independently of regular virus evolution, that is generation of new mutations (mostly by RT errors during the reverse transcription process) and selection of beneficial changes.

## PBS evolution differs from regular virus evolution

We realize that there may be a fitness difference between tRNA^Lys3^- and tRNA^Lys5^-using viruses for several reasons. These tRNA molecules may have a different intracellular concentration, which is supported by a recent deep sequencing study that revealed 1966 tRNA^Lys3^ sequence reads versus 53 tRNA^Lys5^ reads, yielding a 37:1 ratio ([17] and unpublished results). These tRNAs may be differentially packaged in the virion particle or differentially recognized by the viral RT enzyme [18, 19]. However, it is not possible to measure the replication fitness of these two pure virus populations as PBS-mixtures will be generated upon the first round of reverse transcription. The rapid reversion of the PBS^Lys5^ virus to the PBS^Lys3^ sequence is caused by frequent tRNA^Lys3^ annealing at PBS^Lys5^ and should not be interpreted as a large fitness impact. In other words, the special nature of the PBS-tRNA axis means that it cannot be compared to other genome segments in evolutionary terms and in fitness calculations. These insights also explain the ‘surprising’ result of rapid reversion in the absence of continuous virus replication [2]. No continuous virus replication is needed to correct the PBS mutation as a single reverse transcription event will do the job.

## No need to implement complex DNA repair, the primer tRNA rules

There is no need to implement a DNA repair action to explain the rapid reversion as this occurs by proviral DNA strand segregation upon cell division. There is also no need to introduce premature termination of reverse transcription to explain progeny proviruses with a mutant PBS, as this is caused by tRNA^Lys5^ annealing. Such premature termination of reverse transcription—before the mismatching nucleotide in the tRNA primer or viral PBS is copied, which corresponds to maximally 7 tRNA nucleotides or 10 PBS nucleotides—seems unlikely anyway as the remaining complementarity between the PBS copy and the tRNA copy will not likely allow an efficient second strand transfer during reverse transcription.

## Conclusions

We focused exclusively on the PBS mutation that was corrected in SIVmac239Opt. Rapid reversion of this mutation upon SIVmac239 replication was mistakenly interpreted as indicating a major impact on virus replication. The other three correcting mutations in SIVmac239Opt represent more regular positions in the viral genome and reversion of these mutations in long-term SIVmac239 cultures may indeed correlate with an fitness impact.
